# On Exalgine as an Analgesic

**Published:** 1891-05

**Authors:** C. Ferreira

**Affiliations:** Rio De Janeiro


### ON EXALGINE AS AN ANALGESIC.

BY DR. C. FERREIRA, RIO DE JANEIRO.

*(Translated from the Bull. Gin. de Therapeutique.)*

From the cases in which I have had occasion to use exalgine, I select five, as of particular interest, and which confirm the results obtained by Dujardin-Beaumetz, Gaudineau, Frazer, etc.

CASE I. *Locomotor Ataxia.*—J., aged 46, fair constitution no nervous antecedents; had syphilis in 1883; April, 1890. I established the fundamental symptoms of tabes: Disturbance of tactile sensibility, girdle pains, lightning pains, absolute abolition of the rotulian reflexes, Romberg's sign, etc. I ordered five grains of exalgine daily with prompt amelioration, and at the end of three days the lightning pains had entirely disappeared. Later on these again appeared, but with less intensity and were again controlled by the same remedy.

CASE II. *Intense Intercostal Neuralgia.*—Mrs. C., June 12, 1890. Is very anæmic and has hysteric and neuralgic troubles. Complained of very severe pain for two days. There were no respiratory nor circulatory difficulties. I prescribed exalgine, (6 grs.) one-half at night and the rest in the morning. The pain, much amended by the first dose, disappeared entirely after the second, and has not since recurred.

CASE III. *Facial Neuralgia.*—Girl of 12 years. Had taken a variety of medicines, ending with antipyrin, without striking results. Two doses of exalgine of 3 grs. each, two hours apart, completely cured the neuralgia.

CASE IV. *Acute Articular Rheumatism.*—A man of 26, who suffered from rheumatism of the elbow and wrist to such an extent that movements of these articulations caused him to cry out. Topical applications had given no relief.

I ordered 40 cgrm. (6 grs.) daily in two doses, with the result of almost immediate relief. Complete cure of the pain followed in a few days with subsidence of the swelling of the joints.

CASE V. *Angina Pectoris.*—Negress, 40 years, addicted to alcoholic excess. Suffered greatly from thoracic pains. I found all the symptoms of a generalized arterio-sclerosis, with the physical signs of dilation of the aorta. Exalgine effected a rapid cure of the pain in this case.

The above facts show clearly the value of exalgine in the symptom of pain, and the drug should be more widely known. Tolerance of the drug was perfect, and I have never remarked any disagreeable effect.

